# Tuscan black kale sprout extract bioactivated with myrosinase: a novel natural product for neuroprotection by inflammatory and oxidative response during cerebral ischemia/reperfusion injury in rat

**DOI:** 10.1186/s12906-015-0929-4

**Published:** 2015-11-06

**Authors:** Sabrina Giacoppo, Maria Galuppo, Gina Rosalinda De Nicola, Renato Iori, Placido Bramanti, Emanuela Mazzon

**Affiliations:** IRCCS Centro Neurolesi “Bonino-Pulejo”, Via Provinciale Palermo, contrada Casazza, 98124 Messina, Italy; Consiglio per la ricerca in agricoltura e l’analisi dell’economia agraria, Centro di ricerca per le colture industriali (CRA-CIN), Via di Corticella 133, 40128 Bologna, Italy

**Keywords:** Glucosinolates, *R*_*S*_-(−)-glucoraphanin, *R*-Sulforaphane, Blood–brain barrier, Oxidative stress, Apoptosis

## Abstract

**Background:**

Cerebral ischemia and reperfusion (CIR) is a pathological condition characterized by a first blood supply restriction to brain followed by the consequent restoration of blood flow and simultaneous reoxygenation.

The aim of this study was to evaluate the neuroprotective effects of Tuscan black kale sprout extract (TBK-SE) bioactivated with myrosinase enzyme, assessing its capability to preserve blood–brain barrier (BBB), in a rat model of CIR.

**Methods:**

CIR was induced in rats according to a classic model of carotid artery occlusion for a time period of 1 h and the reperfusion time was prolonged for seven days.

**Results:**

By immunohistochemical evaluation and western blot analysis of brain and cerebellum tissues, our data have clearly shown that administration of bioactive TBK-SE is able to restore alterations of tight junction components (claudin-5 immunolocalization). Also, bioactive TBK-SE reduces some inflammatory key-markers (p-selectin, GFAP, Iba-1, ERK1/2 and TNF-α), as well as the triggering of neuronal apoptotic death pathway (data about Bax/Bcl-2 balance, p53 and cleaved-caspase 3) and the generation of radicalic species by oxidative stress (results focused on iNOS, nitrotyrosine and Nrf2).

**Conclusion:**

Taken together, our findings lead to believe that bioactive TBK-SE exerts pharmacological properties in protecting BBB integrity through a mechanism of action that involves a modulation of inflammatory and oxidative pathway as well into control of neuronal death.

## Background

Cerebral ischemic stroke regards for approximately 80 % of all strokes [1] and often it results from the occlusion of a cerebral artery caused by a thrombus or embolus that leads to an immediate loss of the normal intake of oxygen and glucose to cerebral tissues [2].

Ready initiation of reperfusion is the most effective treatment for reducing infarct area and behavioural deficits caused by ischemia. Paradoxically, however, blood flow restoration is causative of additional injury during the cascade of events that characterize and identify the so-called cerebral ischemia/reperfusion (CIR) injury [3].

It has been widely demonstrated that excitotoxicity, ionic imbalance, adhesion molecules upregulation, reactive oxygen and nitrogen species (ROS/RNS) formation, inflammation and apoptosis are the main mechanisms involved in CIR [4, 5]. It is well known also that all these events contribute to blood–brain barrier (BBB) breakdown, considered as a critical step in cerebral ischemia pathogenesis [6].

BBB integrity and maintenance of homeostasis in central nervous system (CNS) are critically dependent of tight junctions (TJs) between cerebrovascular endothelial cells. Any abnormality in the structure or function of TJs can lead to BBB dysfunction that consequently may contribute to the development of neurological damage [6].

Several experimental data showed that oxidative stress may affect TJ components of BBB through the activation of several pathways [7]. In fact, during CIR, production of ROS is dramatically increased and involves endogenous antioxidant systems leading to oxidative stress and ultimately contributing to neuronal cell death [8].

For this reason, antioxidants have been the focus of studies for developing neuroprotective drugs to be used in cerebral ischemia treatment. To date there is no clinically effective therapy for stroke management except tissue-plasminogen activator (t-PA) [9].

In this scenario, growing interest has been focused on the neuroprotective properties of natural compounds and glucosinolates (GLs), a group of secondary metabolites found in Brassica vegetables (Cruciferae), in helping to maintain human health [10]. Increased consumption of cruciferous vegetables has been associated with a decreased risk of several cardiovascular and neurodegenerative diseases [11–13]. In specific, the hydrolysis of GLs by the plant enzyme myrosinase (Myr) results in the formation of corresponding biologically active compounds, the isothiocyanates (ITCs) [14]. To date, more than 120 GLs have been identified in plant [15]. Among them, one of the most studied is *R*_*S*_-(−)-glucoraphanin [GRA; 4(*R*_*S*_)-methylsulfinylbutyl glucosinolate] a thiosaccharidic compound found notably in Tuscan black kale (*Brassica oleracea* L. var. acephala sabellica), for its numerous properties as antinflammatory as well as antioxidant agent, especially for neurodegenerative diseases treatment [12, 16–18].

In the light of these recent findings, the purpose of our study was to investigated whether a freeze-dried Tuscan black kale sprouts extract containing about 15 % of GRA and other minor GLs and bioactivated with Myr (bioactive TBK-SE) has neuroprotective effects in a chronic experimental model of CIR. Also, we investigated the possible neuroprotective role of bioactive TBK-SE, as a novel important field of action potentially applicable in BBB dysfunctions through a repair mechanism at the level of TJs proteins and thus, the progression of neurological injury.

Finally, other important aim of this study was to suggest this natural extract as a promising source of alternative medicine for the prevention and/or treatment of cerebral ischemia. In addition, as being a natural phytochemical, we believe that bioactive TBK-SE could be introduced as an herbal medicine without adverse effects, at least in association with current conventional therapies.

## Methods

### Plant source and extract preparation

Ripe seeds of Tuscan black kale (*Brassica oleracea* (L.) ssp acephala (DC) var. Sabellica L. cv. 0D74) were supplied by Suba Seeds Company (Longiano, FC, Italy) and stored in a dry and dark place at room temperature. Seeds were identified by a lot number and guaranteed by the producer for the quality and the homogeneity of the product. Seeds were surface sterilised by soaking for 30 min in 1 % sodium hypochlorite and rinsed with tap water. Sprouts were grown at room temperature by using an automatic sprouter VitaSeed (Suba Seeds, Longiano, FC, Italy) under an 8 h/16 h light/dark cycle. Four-day old sprouts were gently washed with tap water, whole frozen, freeze-dried and ground to a fine powder. Fine powdered freeze-dried sprouts (30 g) were extracted in boiling 70 % (v/v) ethanol (800 ml) for 5 min at 80 °C using an Ultra-Turrax T25 homogenizer (IKA-Werk, Staufen, Germany), and then centrifuged with a J2-MC centrifuge (Beckman, Palo Alto, CA, USA) at 17,700 g for 40 min at 10 °C. The solid residue was extracted a second time with the same w/v ratio and centrifuged as before. The two supernatants were collected and the volume was reduced three fold in a rotary evaporator at a temperature of 40 °C. The concentrated extract was kept in an ice bath overnight. Precipitated proteins were removed by centrifugation, and finally the extract was freeze-dried (DLAB 500, Italian Vacuum Technology).

### Determination of glucosinolate content

TBK-SE was analysed for GL profile and content according to the EU official ISO 9167–1 method [19] which is based on the HPLC analysis of desulfo-GL, as previously described [20]. Eight independent HPLC determinations were performed.

### Myrosinase purification

The enzyme myrosinase (Myr) was isolated from seeds of *Sinapis alba* L. according to a reported method with some modifications [21]. Briefly, the enzyme was extracted from white mustard seeds with water and purified by affinity chromatography on Con A-Sepharose. Then, the active fractions coming from affinity chromatography were pooled and dialyzed against 50 mM phosphate buffer pH 6.5 containing 0.15 M NaCl. The dialyzed Myr solution was concentrated and loaded into a prepacked Superdex 200 HiLoad 26/60 gel filtration column (GE Healthcare) equilibrated with 50 mM phosphate buffer pH 6.5 containing 0.15 M NaCl connected with a fast protein liquid chromatography system (AKTA FPLC System, GE Healthcare, Milan, Italy). The active fractions were pooled and concentrated by Millipore Amicon Stirred Cell Model 8400 using a UF membrane 30 KDa MWCO (Millipore). The stock solution used in the present study had a specific activity of 60 units/mg of soluble protein. The enzymatic activity was 32 U/ml and the solution was stored at 4 °C in sterile saline solution at neutral pH until use. One Myr unit was defined as the amount of enzyme able to hydrolyze 1 μmol/min of sinigrin at pH 6.5 and 37 °C.

### Animals

Male Sprague–Dawley rats (about 9 weeks old) (Harlan, Italy) 200–250 g weight were used. Rats were housed in a controlled environment and provided with standard rodent chow and water. Animal care was in compliance with Italian regulations on protection of animals used for experimental and other scientific purposes (D.M. 116/92) as well as with the EEC regulations (O.J. of E.C.L 358/1 12/18/1986).

### Ethics statement

This study was carried out in strict accordance with the recommendations in the guide for the care and use of laboratory animals of the National Institutes of Health. The protocol was approved by the Ministry of Health “General Direction of animal health and veterinary drug”. In particular, animal care was in compliance with Italian regulations on protection of animals used for experimental and other scientific purposes (D.M. 116/92) as well as with the EEC regulations (O.J. of E.C.L 358/1 12/18/1986). Also, it was minimized number of animals used for this experiment and their suffering.

### Cerebral ischemia/reperfusion (CIR) induction

After anaesthesia induced with an anaesthetic cocktail composed of tiletamine plus xylazine (1 ml/Kg i.p.), CIR was induced in rats according to a standardized method already used in our previous published study of 2014 [22] that originally was described by Awooda et al. [23] making some modifications. In brief, in the supine position, a midline ventral incision was made in the neck of each animal; the left carotid artery was exposed, separated from the vagus nerve and occluded for 1 h by clamping with small vascular clips and by inducing hypotension to generate a cerebral ischemia animal model. A phase of reperfusion of blood flow of the duration of seven days was followed.

Blood pressure was continuously monitored through a blood pressure recorder (Ugo Basile, Varese, Italy), a non invasive method that allows to check on a display the systolic and diastolic blood pressure of rat during the surgical procedures by the application of a tail cuff. This allowed to ascertaining the reduction of blood flow following carotid artery occlusion and the increasing after blood flow restoration. In specific, before the start surgical procedures, it was recorded a baseline blood pressure value of about 108 ± 5 mmHg in rats and a blood pressure value of about 49 ± 5 mmHg immediately after the clamping, indicating that cerebral ischemia was successful induced. Following removal of vascular clip, blood pressure returned to the value of about 108 ± 5 mmHg.

In addition, during the observation period of seven days, we have recorded eyelid edema associated to hemorrhagic lachrymation in animal subjected to CIR, further indicating that there had been alteration in cerebral blood flow circulation.

### Myrosinase bioactivation of TBK-SE and animal treatment

TBK-SE was dissolved in PBS solution pH 7.2 at room temperature and before rat i.p. treatment, the action of Myr (17 mg TBK-SE, containing 2.6 mg GRA)/rat plus 20 μl Myr enzyme/1 ml) for 15 min at 37 °C allowed having bioactive TBK-SE quickly.

### Experimental groups

Rats were randomly allocated into the following groups (*N* = 20 total animals):

- **Untreated CIR group**: rats were subjected to 1 h of carotid artery occlusion followed by 7 days of reperfusion (*N* = 10);
- **Bioactive TBK**-**SE**-**treated CIR group**: rats were subjected to surgical procedures described as above and bioactive TBK-SE (17 mg TBK-SE/rat plus 20 μl Myr enzyme/1 ml) was administered 15 min after ischemia and daily for seven days (*N* = 10).

Among the experimental groups, “Myr-control” group has not been provided because in our previous studies it was tested that Myr injection alone was without effects. As shown, Myr injection did not display allergic effect as no eosinophil cells were detected by hematocrit analysis [22].

At the end of the experiment, blood was collected by cardiac puncture and animals were euthanized. Brain and cerebellum tissues were sampled and processed in order to perform morphological evaluation and molecular biology analysis.

### Immunohistochemical evaluation

At 7 days following CIR-induction, brains were sampled and fixed in 10 % (w/v) PBS-buffered formaldehyde and 7 μm sections were prepared from paraffin-embedded tissues. After deparaffinization with xylene, sections of brain samples were hydrated in graded ethanol. Detection of claudin-5, p-selectin, GFAP and iNOS, Nitrotyrosine, Nrf2 and Bax was carried out after boiling in citrate buffer 0.01 M pH 6 for 4 min. Endogenous peroxidase was quenched with 0.3 % (v/v) hydrogen peroxide in 60 % (v/v) methanol for 30 min. Nonspecific adsorption was minimized by incubating the section in 2 % (v/v) normal goat serum in PBS for 20 min.

Sections were incubated overnight with:

- anti-Claudin-5 monoclonal antibody (1:100 in PBS v/v; Novus Biologicals);
- anti-p-selectin polyclonal antibody (1:100 in PBS v/v; Santa Cruz Biotechnology, Inc.);
- anti-GFAP monoclonal antibody (1:50 in PBS v/v; Cell Signaling Technology);
- anti-iNOS polyclonal antibody (1:100 in PBS v/v; Santa Cruz Biotechnology, Inc.);
- anti-Nitrotyrosine polyclonal antibody (1:1000 in PBS v/v; Millipore);
- anti-Nrf2 polyclonal antibody (1:100 in PBS v/v; Santa Cruz Biotechnology, Inc.);
- anti-Bax polyclonal antibody (1:100 in PBS v/v; Santa Cruz Biotechnology, Inc.).

Endogenous biotin or avidin binding sites were blocked by sequential incubation for 15 min with biotin and avidin (DBA, Milan, Italy), respectively. Sections were washed with PBS and incubated with secondary antibody. Specific labelling was detected with a biotin-conjugated goat anti-rabbit IgG and avidin–biotin peroxidase complex (Vectastain ABC kit, VECTOR). The counterstain was developed with peroxidase substrate kit DAB (brown colour) or DAB nickel solution addicted (black colour) (Vector Laboratories, Inc.) and Hematoxylin (blue background) or nuclear fast red (Vector Laboratories, Inc.). To verify the binding specificity, some sections were also incubated with only the primary antibody (no secondary) or with only the secondary antibody (no primary). In these cases no positive staining was found in the sections, indicating that the immunoreaction was positive in all the experiments carried out.

All sections were obtained using light microscopy (LEICA DM 2000 combined with LEICA ICC50 HD camera). To perform densitometric analysis, quantitative data were carried out using Leica Application Suite V4.2.0 software.

### Western blot analysis

All the extraction procedures were performed on ice using ice-cold reagents. In brief, cerebellum tissues were suspended in extraction buffer containing 0.32 M sucrose, 10 mM Tris–HCl, pH 7.4, 1 mM EGTA, 2 mM EDTA, 5 mM NaN3, 10 mM 2-mercaptoethanol, 50 mM NaF, protease inhibitor tablets (Roche Applied Science, Monza, Italy), and they were homogenized at the highest setting for 2 min. The homogenates were chilled on ice for 15 min and then centrifuged at 1000 g for 10 min at 4 °C, and the supernatant (cytosol + membrane extract from brain tissue) was collected to evaluate content of citoplasmatic proteins.

The pellets were suspended in the supplied complete lysis buffer containing 1 % Triton X-100, 150 mM NaCl, 10 mM Tris–HCl, pH 7.4, 1 mM EGTA, 1 mM EDTA protease inhibitors (Roche), and then were centrifuged for 30 min at 15.000 g at 4 °C. Then, supernatant containing nuclear extract was collected to evaluate the content of nuclear proteins. Supernatants were stored at −80 °C until use. Protein concentration in homogenate was estimated by Bio-Rad Protein Assay (Bio-Rad, Segrate, Italy) using BSA as standard, and 20 μg of cytosol and nuclear extract from each sample were analyzed.

Proteins were separated on sodium dodecyl sulfate-polyacrylamide minigels and transferred onto PVDF membranes (Immobilon-P Transfer membrane, Millipore), blocked with PBS containing 5 % nonfat dried milk (PM) for 45 min at room temperature, and subsequently probed at 4 °C overnight with specific antibodies for Phospho-p44/42 MAPK (ERK1/2) (1:2000; Cell Signaling Technology), Bcl-2 (1:500; Cell Signaling Technology), Bax (1:500; Cell Signaling Technology), Nrf2 (1:100; Cell Signaling Technology), Nitrotyrosine (1:2000; Millipore), Iba-1 (1:1000; Abcam), p53 (Abcam 1:2000;) and cleaved-caspase 3 (1:500; Cell Signaling Technology), in 1x PBS, 5 % (w/v) non fat dried milk, 0.1 % Tween-20 (PMT).

HRP-conjugated goat anti-rabbit IgG or goat anti-mouse IgG were incubated as secondary antibodies (1:2000; Santa Cruz Biotechnology, Inc.) for 1 h at room temperature.

To ascertain that blots were loaded with equal amounts of protein lysates, they were also incubated with antibody for GAPDH HRP Conjugated (1:1000; Cell Signaling Technology), p42 MAP Kinase (Erk 2) (1:1000; Cell Signaling Technology) and beta-actin (1:1000; Santa Cruz Biotechnology, Inc). The relative expression of protein bands was visualized using an enhanced chemiluminescence system (Luminata Western HRP Substrates, Millipore) and proteic bands were acquired and quantified with ChemiDoc™ MP System (Bio-Rad) and a computer program (ImageJ software) respectively.

Blots are representative of three separate and reproducible experiments. The statistical analysis was carried out on three repeated blot performed on separate experiments.

### Blood sampling

At the sacrifice, blood samples were collected via cardiac puncture in Serum Separator Tubes (Vacutainer® SSTTM II Advance, BD Diagnostic, Milan, Italy) and centrifuged following at least 30 min from the collection at 2000 g speed for 10 min. The achieved serum was collected, aliquoted and stored at −80 °C to be used in next investigations.

### TNF-α assay

ELISA kit for TNF-α parameter assay (R&D system Europe, Ltd, Abingdon, UK) was purchased to detect TNF-α levels in serum samples. The kit was used according to the manufacturer’s instruction and achieved O.D. were tabulated and analyzed using a software of elaboration data.

### Statistical analysis

Data were analyzed in GraphPad Prism version 6.0 (GraphPad Software, La Jolla, CA). The results were analyzed by unpaired Student’s *t*-test. A *p* value of < 0.05 was considered to be statistically significant. All results achieved are presented as the means ± S.E.M. of n experiments.

## Results

### Glucosinolate content in TBK-SE and myrosinase bioactivation

**Fig. 1:**
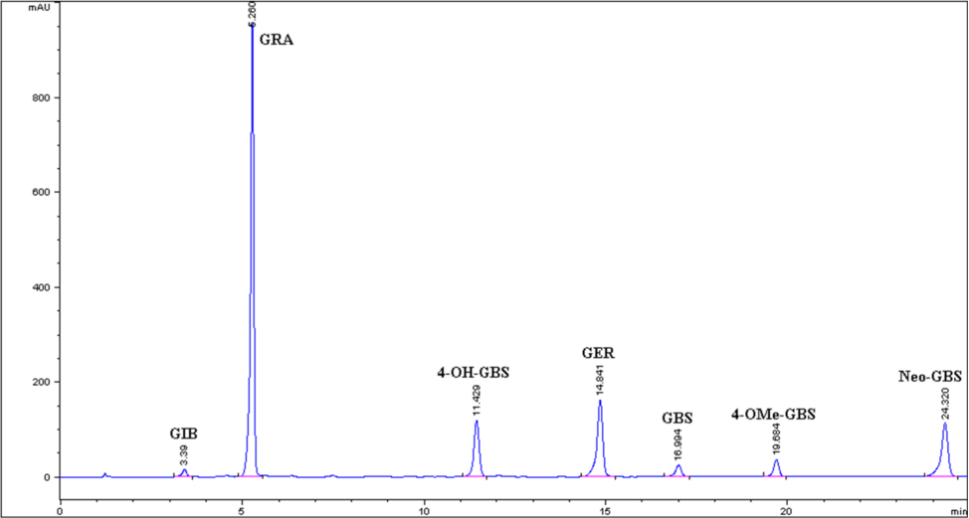
HPLC chromatogram of glucosinolates (GLs) isolated from Tuscan black kale (*Brassica oleracea* (L.) ssp acephala (DC) var. Sabellica L. cv. 0D74) sprout extract. GIB, glucoiberin; GRA, *R*_*S*_-(−)-glucoraphanin; GER, glucoerucin; 4-OH-GBS, 4-hydroxy glucobrassicin; GBS, glucobrassicin, 4-OMe-GBS, 4-methoxy glucobrassicin; Neo-GBS, neoglucobrassicin

Individual and total content of GLs in TBK-SE are reported in Table 1, and Fig. 1 shows a typical HPLC chromatogram of GLs present in the extract (Fig. 1). The extract resulted to be highly enriched in GLs containing 21.1 % (w/w) of total GLs. The most abundant were two aliphatic GL with sulfur-containing side chain, with predominant GRA, the precursor of *R*-sulforaphane, followed by glucoerucin (GER, 4-methylsulfanylbutyl GL) the precursor of erucin, representing 72 % and 17 % of total GLs, respectively. It is worth noting that, TBK-SE was free of the goitrogenic progoitrin ((2*S*)-2-hydroxy-3-butenyl GL) and contained only limited amount of indole GLs, 0.98 % (w/w) 4-hydroxy-glucobrassicin (4-OH-GBS, 4-hydroxy-3-indolylmethyl GL), 0.18 % (w/w) glucobrassicin (GBS, 3-indolylmethyl GL), 0.21 % (w/w) 4-methoxy glucobrassicin (4-OMe-GBS, 4-methoxy-3-indolylmethyl GL) and 0.67 % (w/w) neoglucobrassicin (Neo-GBS, N-methoxy-3-indolylmethyl). Treatment of TBK-SE with Myr enzyme catalyzed the quantitative transformation of aliphatic GLs into ITCs, *R*-sulforaphane being the major one, as already described [20], thus indole GLs are known to be hydrolysed into highly unstable ITC and are spontaneously transformed to carbinols.

**Table 1:** Glucosinolate (GL) content of Tuscan black Kale (*Brassica oleracea* (L.) ssp acephala (DC) var. Sabellica L. cv. 0D74) sprout extract

	Aliphatic GLs			Indole-type GLs				Total GLs
	GIB	GRA	GER	4-OH-GBS	GBS	4-OMe-GBS	Neo-GBS	
μmol/g	6.5 ± 0.4	317.2 ± 8.2	79.5 ± 2.8	19.4 ± 1.8	3.7 ± 0.5	4.1 ± 0.2	13.0 ± 0.3	443.4 ± 14.2
mg/g	3.0 ± 0.2	150.9 ± 3.9	36.5 ± 1.3	9.8 ± 0.9	1.8 ± 0.2	2.1 ± 0.1	6.7 ± 0.2	210.8 ± 6.8

The data represent the mean ± SD of two replicates experiments with 4 samples analysed per replicate (*n* = 8)

*GIB* glucoiberin, *GRA R*_*S*_-(−)-glucoraphanin, *GER* glucoerucin, *4-OH-GBS* 4-hydroxy glucobrassicin, *GBS* glucobrassicin, *4-OMe-GBS* 4-methoxy glucobrassicin, *Neo-GBS* neoglucobrassicin

### Bioactive TBK-SE restores BBB vascular endothelium after CIR induction

In order to evaluate whether BBB breakdown is accompanied by the loss or alterations of TJ-associated molecules from the BBB TJs following CIR induction, we investigated the claudin-5 expression by immunohistochemical evaluation.

**Fig. 2:**
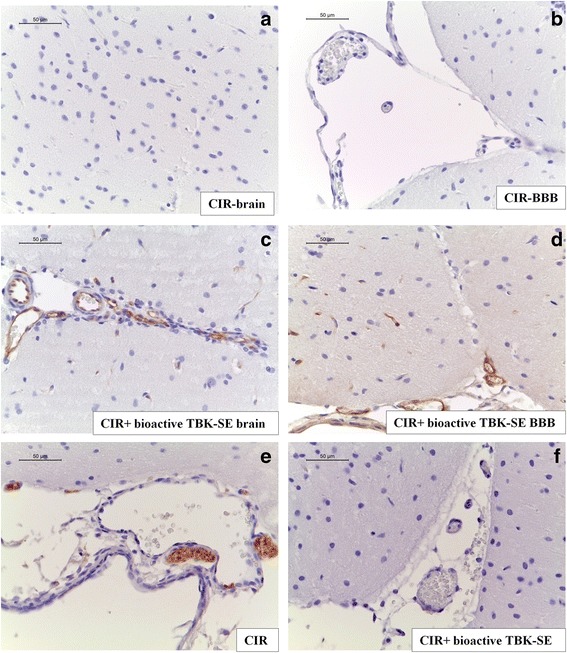
Bioactive TBK-SE restores BBB vascular endothelium. Sections from CIR rats did not show positive staining for claudin-5 in temporal lobe of brain tissue (**a**) as well as at the level of vascular endothelium of BBB in temporal lobe area of the brain (**b**). In contrast, in both two sections obtained from bioactive TBK-SE-treated rats it was observed a normal distribution of claudin-5 (**c** and **d**). Also, immunohistochemical localization of p-selectin displayed an increased expression of adhesion molecules following CIR in the vascular endothelium (**e**), while treatment with bioactive TBK-SE clearly reduced the degree of positive staining for p-selectin in brain tissues (**f**)

**Fig. 3:**
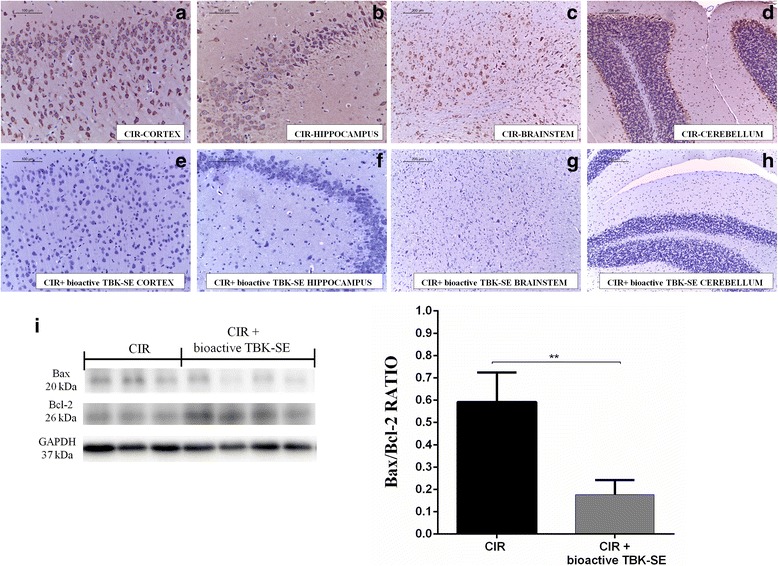
Bioactive TBK-SE treatment modulates unbalance between Bax and Bcl-2. Bax was evaluated by immunohistochemical evaluation in brain sections after CIR induction. Brain sections obtained from CIR untreated rats exhibited positive staining for Bax in cortex (**a**), hippocampus (**b**), brainstem (**c**) and cerebellum (**d**) of CIR rats, while rats treated with bioactive TBK-SE showed negative staining for Bax in cortex (**e**), hippocampus (**f**), brainstem (**g**) and cerebellum (**h**). Panel I shows ratio between Bax and Bcl-2 in cerebellum tissues, showing an higher expression of Bax/Bcl-2 in CIR untreated rats, attenuated by administration of bioactive TBK-SE (**i**). GAPDH was used as internal control. **p < 0.05 *vs* CIR

Sections obtained from CIR rats did not show positive staining for claudin-5 in temporal lobe of brain tissue (Fig. 2a) as well as at the level of vascular endothelium of BBB in temporal lobe area of the brain (Fig. 2b). In contrast, bioactive TBK-SE treatment normalized the positive staining for claudin-5 in different districts, as shown by immunohistochemical localization in brain section and in BBB vascular endothelium of CIR rats (Fig. 2c and d, see densitometric analysis Fig. 3).

Also, immunohistochemical localization of p-selectin showed an increased expression of adhesion molecules following CIR in the vascular endothelium (Fig. 2e), while treatment with bioactive TBK-SE clearly reduced the degree of positive staining for p-selectin in brain tissues (Fig. 2f, see densitometric analysis Fig. 3).

### Bioactive TBK-SE modulates GFAP and Iba-1expression after CIR

**Fig. 4:**
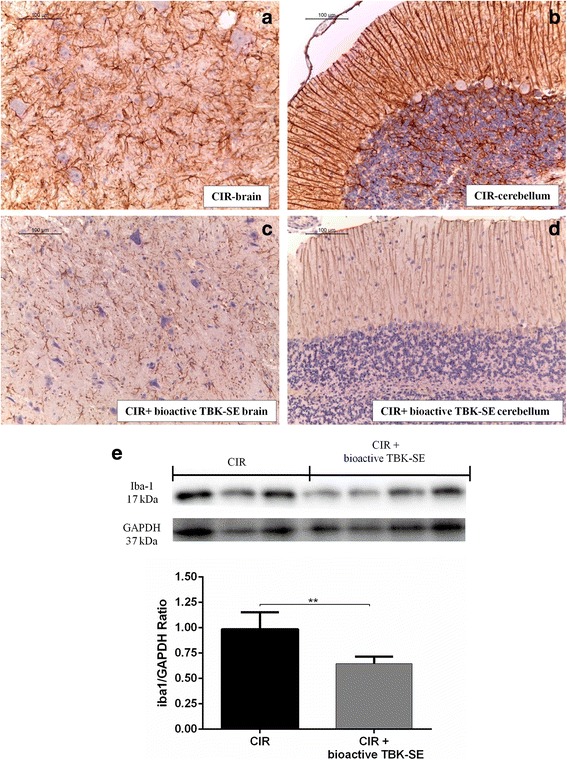
Effects of Bioactive TBK-SE on GFAP and Iba-1 expression. The immunohistochemical analysis for GFAP showed that positive staining for GFAP was observed in the tissues obtained from CIR rats both in brain (**a**) as well as in cerebellum sections (**b**). In contrast, a reduction of GFAP positive staining was evident in bioactive TBK-SE-treated group in both two different areas (**c** and **d**). By western blot analysis it has been shown a significant increase Iba-1 expression in cerebellum samples collected from CIR rats seven days after CIR induction. Conversely, levels of Iba-1 were attenuated by administration with bioactive TBK-SE attenuated Iba-1 levels by approximately 50 % (**e**). GAPDH was used as internal control. **p < 0.05 *vs* CIR

**Fig. 5:**
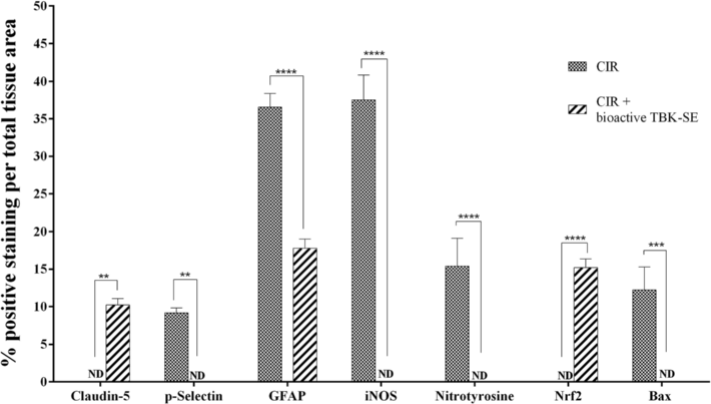
Densitometric analysis for claudin-5, p-selectin, GFAP and i-NOS, nitrotyrosine, Nrf2 and Bax. For immunohistochemical images, densitometric analysis was carried out to quantify and highlight significant differences among experimental groups. *p* value <0.05 was considered significant

Moreover, with the purpose to investigate the cellular mechanisms by which the treatment with bioactive TBK-SE may modulate the astrocyte activation during CIR, we evaluated the GFAP expression by immunohistochemical analysis. GFAP is considered a marker protein for astrogliosis. It was observed a marked positive staining for GFAP in the sections from CIR rats, both in brain (Fig. 4a) and cerebellum sections (Fig. 4b). In contrast, a reduction of GFAP positive staining was evident in pharmacologically treated group (Fig. 4c and d, see densitometric analysis Fig. 5).

Also, western blot analysis showed that Iba-1 levels are substantially increased in cerebellum samples collected from CIR rats seven days after CIR induction, whereas Iba-1 levels were attenuated by approximately 50 % with bioactive TBK-SE administration (Fig. 4e).

### Bioactive TBK-SE regulates iNOS, nitrotyrosine and Nrf2 expression

**Fig. 6:**
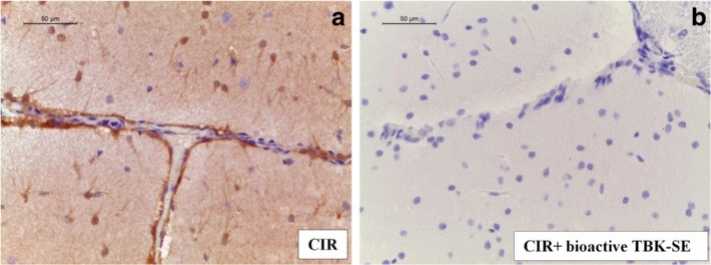
Bioactive TBK-SE modulates production of i-NOS. iNOS was evaluated by immunohistochemical analysis in brain sections 7 days after CIR. Brain sections obtained from CIR rats exhibited positive staining for iNOS (**a**). Bioactive TBK-SE treatment reduced the degree of positive staining for iNOS in lobe temporal area of brain (**b**)

To determine the role of nitric oxide (NO) produced during CIR and to verify whether treatment with bioactive TBK-SE is able to counteract oxidative and nitrosative stress resulting from ischemic damage, we evaluated iNOS and nitrotyrosine expression by immunohistochemical and western blot analysis, after seven days of reperfusion. Immunohistochemical localization of iNOS in temporal lobe area of brain tissues of untreated-CIR rats (Fig. 6a) sampled showed an increased expression of this marker following CIR, while treatment with bioactive TBK-SE significantly reduces the degree of positive staining for iNOS (Fig. 6b, see densitometric analysis Fig. 5).

**Fig. 7:**
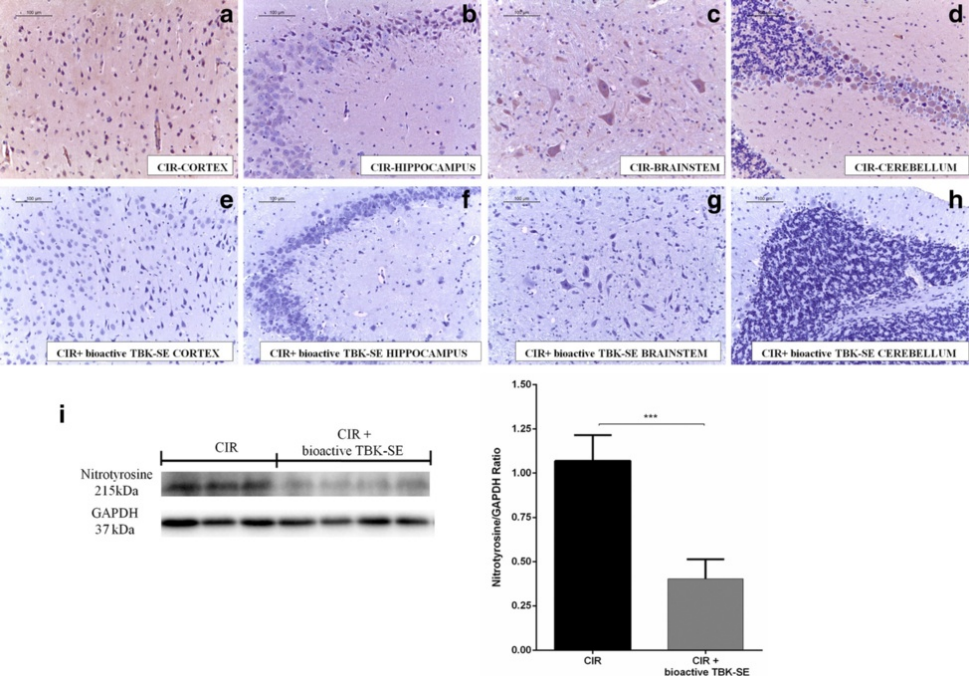
Bioactive TBK-SE modulates nitrotyrosine expression. Nitrotyrosine was evaluated by immunohistochemical evaluation in brain sections after CIR induction. Brain sections obtained from CIR untreated rats exhibited positive staining for nitrotyrosine in cortex (**a**), hippocampus (**b**), brainstem (**c**) and cerebellum (**d**) of CIR rats, while rats treated with bioactive TBK-SE showed negative staining for nitrotyrosine in cortex (**e**), hippocampus (**f**), brainstem (**g**) and cerebellum (**h**). By western blot analysis nitrotyrosine expression was evaluated. It was found a significant increase in nytrotirosine expression in cerebellum samples collected 7 days after CIR-induction from untreated rats. Conversely, cerebellum levels of nytrotirosine were reduced by administration of bioactive TBK-SE (**i**). GAPDH was used as internal control. ***p < 0.05 *vs* CIR

Brain sections obtained from CIR untreated rats exhibited positive staining for nitrotyrosine in cortex (Fig. 7a), hippocampus (Fig. 7b), brainstem (Fig. 7c) and cerebellum (Fig. 7d) of CIR rats, while rats treated with bioactive TBK-SE showed negative staining for nitrotyrosine (Fig. 7e, f, g, h, see densitometric analysis Fig. 5).

In addition, we analyzed cerebellum expression levels of nytrotirosine by western blot analysis. This displayed a significant increase in nytrotirosine expression in cerebellum samples collected 7 days after CIR-induction from untreated rats. Conversely, cerebellum levels of nytrotirosine were reduced by administration of bioactive TBK-SE (Fig. 7i).

**Fig. 8:**
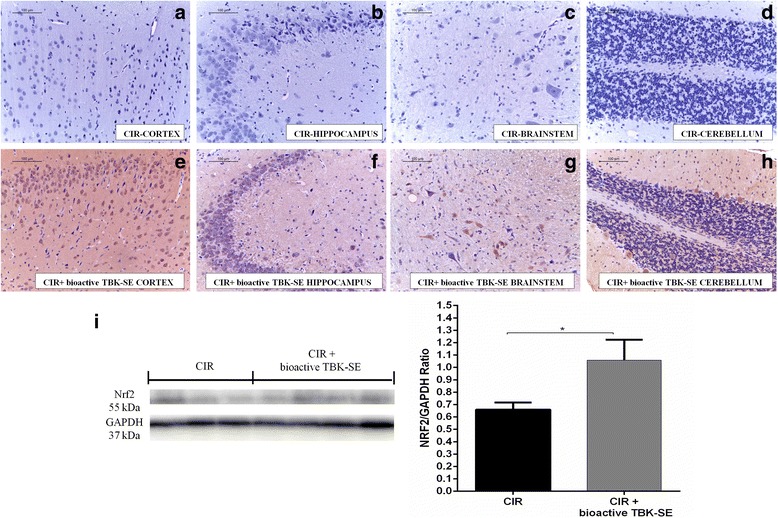
Effects of Bioactive TBK-SE on Nrf2 expression. Negative staining for Nrf2 was observed in cortex (**a**), hippocampus (**b**), brainstem (**c**) and cerebellum (**d**) of CIR rats. On the contrary, positive staining for Nrf2 was observed in cortex (**e**), hippocampus (**f**), brainstem (**g**) and cerebellum (**h**) from rats treated with bioactive TBK-SE. Also, western blot analysis showed a basal level of Nrf2 expression in cerebellum samples obtained from CIR rats. Bioactive TBK-SE treatment significantly increased Nrf2 expression (**i**). GAPDH was used as internal control. *p < 0.05 *vs* CIR

Moreover, it is known that GLs may exert their cytoprotective effects by the ability to induce expression of several enzymes via the Keap1/Nrf2/ARE pathway. Western blot analysis showed a basal level of Nrf2 expression in samples obtained from CIR rats. Treatment of rats with bioactive TBK-SE significantly increased Nrf2 expression () (Fig. 8i). The same result was obtained from immunohistochemical evaluation for Nrf2, showing a positive staining in cortex (Fig. 8a), hippocampus (Fig. 8b), brainstem (Fig. 8c) and cerebellum (Fig.8d) of CIR rats treated with bioactive TBK-SE, and a negative staining in brain of CIR rats (Fig. 8e, f, g, h, see densitometric analysis Fig. 5).

### Effect of bioactive TBK-SE on Phospho-p44/42 MAPK (ERK1/2) expression and TNF-α following CIR

**Fig. 9:**
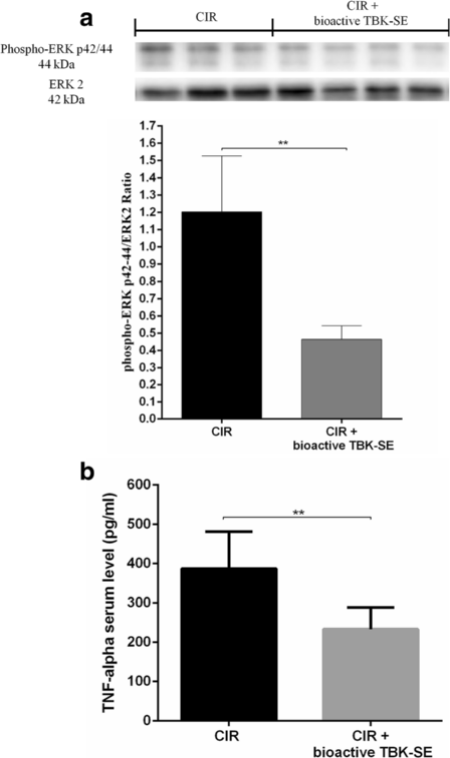
Western blot analysis of ERK1/2 expression and ELISA assay for TNF-α. ERK1/2 expression levels normalized on ERK2 display an increase in rats subjected to CIR, while administration of bioactive TBK-SE reduces levels of ERK1/2 (**a**). ERK2 was used as internal control. **p < 0.05 *vs* CIR. ELISA assay showed that serum levels of TNF-α are significantly higher in untreated CIR rats when compared with TNF-α serum levels of animals treated with bioactive TBK-SE (**b**). **p < 0.05 *vs* CIR

To investigate the cellular mechanisms whereby treatment with bioactive TBK-SE attenuates the development of CIR, we also evaluated the level of ERK1/2 which results in expression of pro-inflammatory genes mediating the inflammatory characteristic of CIR. The activation of MAPK pathways in particular the phosphorylation of ERK1/2 expression was investigated by western blot analysis in cerebellum tissue. ERK1/2 levels were appreciably increased in cerebellum samples taken from rats subjected to CIR, while the treatment of rats with bioactive TBK-SE reduced levels of ERK1/2 (Fig.9a). Also, in order to investigate whether treatment with bioactive TBK-SE can modulate the inflammatory processes triggered by CIR induction through regulating secretion of pro-inflammatory cytokines, the expression levels of TNF-α, serum samples was quantified by ELISA assay. Our results showed that serum levels are significantly higher in untreated CIR rats when compared with serum levels of animals treated with bioactive TBK-SE (Fig. 9b).

### Bioactive TBK-SE treatment inhibits CIR-induced apoptosis

At seven days after CIR, the appearance of proteic effectors of mitochondrial apoptosis, such as pro-apoptotic Bax proteins, was evaluated by immunohistochemical evaluation and western blot. Immunohistochemical evaluation for Bax was performed in different areas of brain tissues. Specifically, a positive staining was found in cortex (Fig. 3a), hippocampus (Fig. 3b), brainstem (Fig. 3c) and cerebellum (Fig. 3d) of CIR rats. On the contrary, treatment with bioactive TBK-SE significantly reduces the degree of positive staining for Bax in all the same regions of the brain (Fig. 3e, f, g, h, see densitometric analysis Fig. 5). Also, by western blot was found that Bax levels were increased substantially in cerebellum tissues from CIR rats. On the contrary, bioactive TBK-SE treatment prevented the CIR-induced Bax expression (Fig. 3i).

Likewise, to detect Bcl-2 expression, extracts from cerebellum tissues of rats were also analyzed by Western blot analysis. A basal level of Bcl-2 expression was detected in samples from CIR rats. Treatment of rats with bioactive TBK-SE significantly attenuated CIR-induced inhibition of Bcl-2 expression (Fig.3i).

**Fig. 10:**
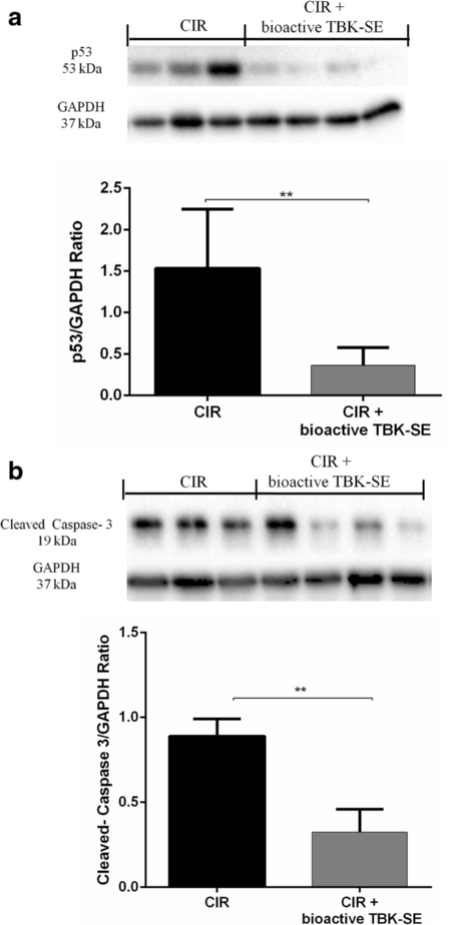
Western blot analysis for p53 and Cleaved-caspase3 expression. p53 expression was detected by western blot analysis. Our data showed a significant expression of p53 in samples collected seven days after CIR-induction. Conversely, levels of p53 were clearly reduced by administration of bioactive TBK-SE (**a**). GAPDH was used as internal control. **p < 0.05 *vs* CIR. By western blot analysis the activation of cleaved-caspase 3 was evaluated. CIR caused a significant increase in cleaved-caspase 3 expression. On the contrary, treatment with bioactive TBK-SE prevented the CIR-induced caspase 3 expression (**b**). GAPDH was used as internal control. **p < 0.05 *vs* CIR

In addition, proteins in the mitochondrial p53 pathway were detected by western blot analysis in cerebellum samples. Our data showed a significant expression of p53 in samples collected seven days after CIR-induction. Conversely, levels of p53 were clearly reduced by administration of bioactive TBK-SE (Fig. 10a).

Finally, sequential activation of caspases plays a central role in the execution-phase of cell apoptosis, leading to programmed cell death by cleavage of cellular substrates. By western blot analysis, we have evaluated the activation of cleaved-caspase 3. Cleaved-caspase 3 levels were appreciably increased in the cerebellum from rats subjected to CIR. On the contrary, treatment with bioactive TBK-SE prevented CIR-induced cleaved-caspase 3 expression (Fig. 10b).

## Discussion

Ischemic stroke is the result of a transient or permanent reduction in cerebral blood flow caused by occlusion of a cerebral artery via an embolus or local thrombosis [24]. After the primary neuronal cell injury, secondary neuronal damage, known as reperfusion injury occurs and exacerbates initial damage [3, 25]. It is well known that cerebral ischemia rapidly raises inflammatory responses in brain, by activating different resident cell populations such as endothelial cells, microglia and astrocytes, as well as inflammatory cytokines release, thereby contributing to BBB breakdown [26, 27]. In fact, BBB disruption is considered as a critical event in the pathogenesis of cerebral stroke. However the molecular mechanisms involved are not completely understood [6].

Among various components of the BBB, the tight junction (TJs) protein claudins are the most widely studied, which are critical for maintaining the BBB structural integrity and permeability. The disruption of the cerebrovascular claudin-5 has been strongly correlated with the dynamic event of BBB breakdown after cerebral ischemia [28, 29]. Immunohistochemical evaluation was performed to demonstrate that CIR induced changes in claudin-5 expression and as well as bioactive TBK-SE is able to control TJs permeability, modulating claudin-5 expression.

Following an impairment of BBB, peripheral leukocytes infiltrate into the brain and the normally immune-privileged cerebral environment is exposed to systemic responses that further aggravate inflammation and brain injury [30].

According to Jin R. et al. [31], our data have revealed an increased expression of p-selectin, an adhesion molecule that stimulates rolling of leukocytes and other inflammatory cell infiltration following induced damage, demonstrating that it was modulated by bioactive TBK-SE administration. With regard to the untreated rats the up-regulation of p-selectin seems to reflect both increased expression of the endothelial cells and the binding of p-selectin positive platelets to the vessel wall, leading in turn an exacerbation of the neuroinflammation status [32].

In addition, we evaluated expression of Iba-1, a novel calcium-binding protein that plays an important role in regulation of microglia function, in which it is specifically expressed [33]. Also, it was found that Iba-1 expression is up-regulated in microglia following cerebral ischemia [33]. Indeed, according to Ohsawa et al. [34], seems that Iba-1 is involved in the Rho family of small GTPase, Rac, and calcium signaling pathways and may be required for cell mobility and phagocytosis of microglia/macrophages. Once activated, microglia develops macrophage-like capabilities including cytokine production, antigen presentation and the release of matrix metalloproteinases that weaken the BBB [35]. Our data confirmed an up-regulation of Iba-1 in CIR rats compared with pharmacologically-treated ones. Moreover, looking at proinflammatory cytokine profile classically activated by microglia during CIR, levels of TNF-α result significantly decreased following bioactive TBK-SE treatment.

It was consistently demonstrated that astrocytes the most abundant population of glial cells, are essential for brain homeostasis and maintenance and maturation of the BBB [36–38]. While astrocytes show a good capability repairing in many CNS processes, they are also capable of secreting inflammatory factors such as cytokines and chemokines, which aggravate brain damage [39]. In fact, astrocytes were found to play an important role also in CIR injury [40].

Moreover, the induction of Nrf2-mediated transcription, particularly in astrocytes, has been shown to protect against neurotoxicity from a variety of injuries, such as cerebral ischemia [41, 42]. According to other studies reported in literature, Nrf2 could play a protective action in astrocytes, decreasing GFAP expression probably through the mechanism related to the glutathione activity [43, 44]. Our results confirmed that treatment with bioactive TBK-SE in CIR rats leads to an upregulation of Nrf2 expression, while GFAP expression was significantly inhibited. This balance prevents that GFAP expressing astrocytes may regulate the integrity of BBB, damaging TJ components and interfering with the normal astrocyte interactions [45–47]. Probably, astrocyte specific Nrf2-mediated protection due to treatment with bioactive TBK-SE could have beneficial effects in counteracting the damage after CIR and this could be associated with the production of several growth factors that may protect neurons from damage.

The local accumulation of NO is also involved in the inflammatory cascade after cerebral ischemia [48, 49]. This mediator enhances cell adhesion molecules expression on endothelial cells and promotes adhesion and transendothelial migration of immune cells [50]. The role of iNOS in ischemia is yet controversial, it was demonstrated that has a beneficial role as modulator or messenger but during oxidative stress condition it is potentially toxic [51]. In fact, over-production of NO through iNOS causes accentuated lipid peroxidation, protein and DNA modifications that result in cellular damage [52]. Our study demonstrates that bioactive TBK-SE reduces the expression of iNOS in tissues from CIR treated rats. Likewise our results demonstrated that bioactive TBK-SE reduced the generation of reactive species through the evaluation of nitrotyrosine expression, chosen as an indirect marker of peroxynitrite activity.

MAP kinase (MAPKs) pathway, investigated through detection of ERK1/2 expression, resulted upregulated in CIR-related mechanisms of pathology but attenuated by bioactive TBK-SE administration.

Although it has been demonstrated that ERK1/2 is a pro-survival factor in the MAP kinase family and contributes to the regulation of cell proliferation and differentiation, under some circumstances, it can function in a pro-apoptotic manner in the neuronal system [53, 54].

Protective effects of bioactive TBK-SE in counteracting apoptosis are evaluable looking to the main apoptosis-regulatory genes, such Bax and Bcl-2.

The changes in the Bax to Bcl-2 ratio have also been studied in several experimental ischemic models proving that excess of Bcl-2 promotes cell survival, while Bax excess induces cell death. Our data showed an up-regulation of Bcl-2 and a downregulation of Bax in pharmacologically-treated rats.

The transcription factor and mediator of apoptosis p53 was also found to be upregulated following stroke [55]. p53 is able to induce apoptosis both by controlling translation of pro-apoptotic p53-checked mediators and by non-transcriptional mechanisms [56], including upregulation of pro-apoptotic Bax and downregulation of Bcl-2 [57, 58].

In specific, according to Leker at al [55], translocation of resident p53 into the nucleus is an early event in p53-induced apoptosis in ischemic brain cells and that the prevention of this early translocation could reduce brain damage. Our data showed an increased nuclear expression in brain ischemic tissues, on the contrary attenuated by treatment with bioactive TBK-SE.

Also, supporting above cited results and adding further evidences about effects of bioactive TBK-SE, we found a modulated cleaved-caspase 3 activity in CIR pharmacologically treated rats.

A protective effect of bioactive TBK-SE suggests that this treatment could interfere with the CIR-induced neuronal death, preserving cells by the injury.

## Conclusion

Alternative medicine is an interesting research field in to discovering potential active substances found in nature for a wide range of applications. As a consequence, our efforts were recently focused to provide new candidates, primarily natural product extracts, for the prevention and treatment of neurological diseases. Here, our results show that bioactive TBK-SE could represent a good and effective approach in the treatment of experimental CIR.

This study was designed and performed in light of the results achieved by our research group in a previous paper [22] in which we investigated the neuroprotective effects of *R*_*S*_-(−)-glucoraphanin purified from Tuscan black kale seeds and bioactivated with myrosinase enzyme in an acute experimental model of CIR. Using this model it was clearly demonstrated that the released *R*-sulforaphane is active on central and peripheral nervous system, through mechanisms which involved both the modulation of the inflammatory pathways and the reduction in the activation of cell death by apoptosis. In specific, it was proved that bioactive *R*_*S*_-(−)-glucoraphanin is able to significantly reduce NF-kB translocation and intercellular adhesion molecule 1 (ICAM-1), as well as the triggering of oxidative species generation (iNOS), and neuronal apoptotic death pathway [22].

Finally, the latest findings obtained by chronic experimental model of CIR lead to believe that bioactive TBK-SE exerts pharmacological properties in protecting BBB integrity through a mechanism of action that involves a modulation of the inflammatory and oxidative pathway as well into the control of neuronal death by apoptosis.

In summary, the relevance of the present study consists in the possible use of bioactive TBK-SE, as a novel natural product for the treatment of damage associated with CIR. The benefit could be ascribed to either as an herbal medicine readily available and both as a safe and well tolerated product that could be used in neuroprotection in cerebral ischemia/reperfusion injury, at least in association with current conventional therapies.

## Abbreviations

(2S)-2-hydroxy-3-butenyl GL: progoitrin
4-OH-GBS: 4-hydroxy-3-indolylmethyl GL, 4-hydroxy-glucobrassicin
4-OMe-GBS: 4-methoxy-3-indolylmethyl GL, 4-methoxy glucobrassicin
BBB: blood–brain barrier
CIR: Cerebral ischemia and reperfusion
CNS: central nervous system
GBS: 3-indolylmethyl GL, glucobrassicin
GER: 4-methylsulfanylbutyl GL, glucoerucin
GLs: glucosinolates
GRA: 4(*R*)-methylsulfinylbutyl GL, *R*_*S*_-(−)-glucoraphanin
ICAM-1: adhesion molecule 1
iNOS: inducible nitric oxide synthase
ITCs: isothiocyanates
Myr: myrosinase
Neo-GBS: N-methoxy-3-indolylmethyl, neoglucobrassicin
NO: nitric oxide
RNS: reactive nitrogen species
ROS: reactive oxygen species
TBK-SE: Tuscan black kale sprout extract
TJ: tight junction
t-PA: tissue-plasminogen activator

## References

[1] Feigin VL, Lawes CM, Bennett DA, Anderson CS (2003). Stroke epidemiology: a review of population-based studies of incidence, prevalence, and case-fatality in the late 20th century. Lancet Neurol.

[2] Go AS, Mozaffarian D, Roger VL, Benjamin EJ, Berry JD, Blaha MJ (2014). Heart disease and stroke statistics--2014 update: a report from the American Heart Association. Circulation.

[3] Dong S, Tong X, Li J, Huang C, Hu C, Jiao H (2013). Total flavonoid of Litsea coreana leve exerts anti-oxidative effects and alleviates focal cerebral ischemia/reperfusion injury. Neural Regen Res.

[4] Doyle KP, Simon RP, Stenzel-Poore MP (2008). Mechanisms of ischemic brain damage. Neuropharmacology.

[5] Ritz MF, Curin Y, Mendelowitsch A, Andriantsitohaina R (2008). Acute treatment with red wine polyphenols protects from ischemia-induced excitotoxicity, energy failure and oxidative stress in rats. Brain Res.

[6] Sandoval KE, Witt KA (2008). Blood–brain barrier tight junction permeability and ischemic stroke. Neurobiol Dis.

[7] Lochhead JJ, McCaffrey G, Quigley CE, Finch J, DeMarco KM, Nametz N (2010). Oxidative stress increases blood–brain barrier permeability and induces alterations in occludin during hypoxia-reoxygenation. J Cereb Blood Flow Metab.

[8] Sugawara T, Chan PH (2003). Reactive oxygen radicals and pathogenesis of neuronal death after cerebral ischemia. Antioxid Redox Signal.

[9] Alavijeh MS, Chishty M, Qaiser MZ, Palmer AM (2005). Drug metabolism and pharmacokinetics, the blood–brain barrier, and central nervous system drug discovery. NeuroRx.

[10] Dinkova-Kostova AT, Kostov RV (2012). Glucosinolates and isothiocyanates in health and disease. Trends Mol Med.

[11] Zhang X, Shu XO, Xiang YB, Yang G, Li H, Gao J (2011). Cruciferous vegetable consumption is associated with a reduced risk of total and cardiovascular disease mortality. Am J Clin Nutr.

[12] Giacoppo S, Galuppo M, Iori R, De Nicola GR, Bramanti P, Mazzon E (2014). The protective effects of bioactive (*R*_*S*_)-glucoraphanin on the permeability of the mice blood–brain barrier following experimental autoimmune encephalomyelitis. Eur Rev Med Pharmacol Sci.

[13] Zhao J, Kobori N, Aronowski J, Dash PK (2006). Sulforaphane reduces infarct volume following focal cerebral ischemia in rodents. Neurosci Lett.

[14] Holst B, Williamson G (2004). A critical review of the bioavailability of glucosinolates and related compounds. Nat Prod Rep.

[15] Fahey JW, Zalcmann AT, Talalay P (2001). The chemical diversity and distribution of glucosinolates and isothiocyanates among plants. Phytochemistry.

[16] Giacoppo S, Galuppo M, Iori R, De Nicola GR, Cassata G, Bramanti P (2013). Protective role of (*R*_*S*_)-glucoraphanin bioactivated with myrosinase in an experimental model of multiple sclerosis. CNS Neurosci Ther.

[17] Galuppo M, Giacoppo S, De Nicola GR, Iori R, Mazzon E, Bramanti P (2013). *R*_*S*_-Glucoraphanin bioactivated with myrosinase treatment counteracts proinflammatory cascade and apoptosis associated to spinal cord injury in an experimental mouse model. J Neurol Sci.

[18] Galuppo M, Iori R, De Nicola GR, Bramanti P, Mazzon E (2013). Anti-inflammatory and anti-apoptotic effects of (*R*_*S*_)-glucoraphanin bioactivated with myrosinase in murine sub-acute and acute MPTP-induced Parkinson’s disease. Bioorg Med Chem.

[19] EEC Regulation 1864/90, Enclosure VIII1990.

[20] Melega S, Canistro D, De Nicola GR, Lazzeri L, Sapone A, Paolini M (2013). Protective effect of Tuscan black cabbage sprout extract against serum lipid increase and perturbations of liver antioxidant and detoxifying enzymes in rats fed a high-fat diet. Br J Nutr.

[21] Pessina A, Thomas RM, Palmieri S, Luisi PL (1990). An improved method for the purification of myrosinase and its physicochemical characterization. Arch Biochem Biophys.

[22] Giacoppo S, Galuppo M, Iori R, De Nicola GR, Bramanti P, Mazzon E (2014). (*R*_*S*_)-glucoraphanin purified from Tuscan black kale and bioactivated with myrosinase enzyme protects against cerebral ischemia/reperfusion injury in rats. Fitoterapia.

[23] Awooda HA, Lutfi MF, Sharara GM, Saeed AM (2013). Role of N-Nitro-L-Arginine-Methylester as anti-oxidant in transient cerebral ischemia and reperfusion in rats. Exp Transl Stroke Med.

[24] Pignataro G, Studer FE, Wilz A, Simon RP, Boison D (2007). Neuroprotection in ischemic mouse brain induced by stem cell-derived brain implants. J Cereb Blood Flow Metab.

[25] Manley GT, Fujimura M, Ma T, Noshita N, Filiz F, Bollen AW (2000). Aquaporin-4 deletion in mice reduces brain edema after acute water intoxication and ischemic stroke. Nat Med.

[26] Yilmaz G, Granger DN (2010). Leukocyte recruitment and ischemic brain injury. Neuromolecular Med.

[27] Skaper SD, Giusti P, Facci L (2012). Microglia and mast cells: two tracks on the road to neuroinflammation. Faseb J.

[28] McColl BW, Rothwell NJ, Allan SM (2008). Systemic inflammation alters the kinetics of cerebrovascular tight junction disruption after experimental stroke in mice. J Neurosci.

[29] Nitta T, Hata M, Gotoh S, Seo Y, Sasaki H, Hashimoto N (2003). Size-selective loosening of the blood–brain barrier in claudin-5-deficient mice. J Cell Biol.

[30] Patel AR, Ritzel R, McCullough LD, Liu F (2013). Microglia and ischemic stroke: a double-edged sword. Int J Physiol Pathophysiol Pharmacol.

[31] Jin R, Song Z, Yu S, Piazza A, Nanda A, Penninger JM (2011). Phosphatidylinositol-3-kinase gamma plays a central role in blood–brain barrier dysfunction in acute experimental stroke. Stroke.

[32] Ishikawa M, Cooper D, Russell J, Salter JW, Zhang JH, Nanda A (2003). Molecular determinants of the prothrombogenic and inflammatory phenotype assumed by the postischemic cerebral microcirculation. Stroke.

[33] Ito D, Tanaka K, Suzuki S, Dembo T, Fukuuchi Y (2001). Enhanced expression of Iba1, ionized calcium-binding adapter molecule 1, after transient focal cerebral ischemia in rat brain. Stroke.

[34] Ohsawa K, Imai Y, Kanazawa H, Sasaki Y, Kohsaka S (2000). Involvement of Iba1 in membrane ruffling and phagocytosis of macrophages/microglia. J Cell Sci.

[35] Iadecola C, Anrather J (2011). The immunology of stroke: from mechanisms to translation. Nat Med.

[36] Abbott NJ, Ronnback L, Hansson E (2006). Astrocyte-endothelial interactions at the blood–brain barrier. Nat Rev Neurosci.

[37] Liu Y, Hu J, Wu J, Zhu C, Hui Y, Han Y (2012). alpha7 nicotinic acetylcholine receptor-mediated neuroprotection against dopaminergic neuron loss in an MPTP mouse model via inhibition of astrocyte activation. J Neuroinflammation.

[38] Barreto G, White RE, Ouyang Y, Xu L, Giffard RG (2011). Astrocytes: targets for neuroprotection in stroke. Cent Nerv Syst Agents Med Chem.

[39] Sun RC, Yang SD, Zhou ZY, Shen CL, Shao JF, Liang JB (2009). Pathologic and immunohistochemical study on lethal primary brain stem injury. Zhonghua Bing Li Xue Za Zhi.

[40] Burnstock G (2008). Purinergic signalling and disorders of the central nervous system. Nat Rev Drug Discov.

[41] Narayanan SV, Dave KR, Saul I, Perez-Pinzon MA (2015). Resveratrol Preconditioning Protects Against Cerebral Ischemic Injury via Nuclear Erythroid 2-Related Factor 2. Stroke.

[42] Jing X, Ren D, Wei X, Shi H, Zhang X, Perez RG (2013). Eriodictyol-7-O-glucoside activates Nrf2 and protects against cerebral ischemic injury. Toxicol Appl Pharmacol.

[43] Calkins MJ, Vargas MR, Johnson DA, Johnson JA (2010). Astrocyte-specific overexpression of Nrf2 protects striatal neurons from mitochondrial complex II inhibition. Toxicol Sci.

[44] Vargas MR, Johnson JA (2009). The Nrf2-ARE cytoprotective pathway in astrocytes. Expert Rev Mol Med.

[45] LaPash Daniels CM, Austin EV, Rockney DE, Jacka EM, Hagemann TL, Johnson DA (2012). Beneficial effects of Nrf2 overexpression in a mouse model of Alexander disease. J Neurosci.

[46] Mignot C, Boespflug-Tanguy O, Gelot A, Dautigny A, Pham-Dinh D, Rodriguez D (2004). Alexander disease: putative mechanisms of an astrocytic encephalopathy. Cell Mol Life Sci.

[47] Willis CL (2012). Imaging in vivo astrocyte/endothelial cell interactions at the blood–brain barrier. Methods Mol Biol.

[48] Mitrasinovic OM, Grattan A, Robinson CC, Lapustea NB, Poon C, Ryan H (2005). Microglia overexpressing the macrophage colony-stimulating factor receptor are neuroprotective in a microglial-hippocampal organotypic coculture system. J Neurosci.

[49] Awooda HA, Lutfi MF, Sharara GGM, Saeed AM (2015). Oxidative/nitrosative stress in rats subjected to focal cerebral ischemia/reperfusion. Int J Health Sci (Qassim).

[50] Kubes P, Suzuki M, Granger DN (1991). Nitric oxide: an endogenous modulator of leukocyte adhesion. Proc Natl Acad Sci U S A.

[51] Sekhon B, Sekhon C, Khan M, Patel SJ, Singh I, Singh AK (2003). N-Acetyl cysteine protects against injury in a rat model of focal cerebral ischemia. Brain Res.

[52] Montalto MC, Hart ML, Jordan JE, Wada K, Stahl GL (2003). Role for complement in mediating intestinal nitric oxide synthase-2 and superoxide dismutase expression. Am J Physiol Gastrointest Liver Physiol.

[53] Cheung ECC, Slack RS (2004). Emerging role for ERK as a key regulator of neuronal apoptosis. Sci STKE.

[54] Lu Z, Xu S (2006). ERK1/2 MAP kinases in cell survival and apoptosis. IUBMB Life.

[55] Leker RR, Aharonowiz M, Greig NH, Ovadia H (2004). The role of p53-induced apoptosis in cerebral ischemia: effects of the p53 inhibitor pifithrin alpha. Exp Neurol.

[56] Sheikh MS, Fornace AJ (2000). Role of p53 family members in apoptosis. J Cell Physiol.

[57] Cregan SP, MacLaurin JG, Craig CG, Robertson GS, Nicholson DW, Park DS (1999). Bax-dependent caspase-3 activation is a key determinant in p53-induced apoptosis in neurons. J Neurosci.

[58] Xiang H, Kinoshita Y, Knudson CM, Korsmeyer SJ, Schwartzkroin PA, Morrison RS (1998). Bax involvement in p53-mediated neuronal cell death. J Neurosci.

